# The Oxidative Stress of Human Sperm Cryopreservation

**DOI:** 10.3390/antiox14040402

**Published:** 2025-03-28

**Authors:** Steven D. Fleming, Laura K. Thomson

**Affiliations:** 1 Discipline of Anatomy & Histology, School of Medical Sciences, University of Sydney, Sydney, NSW 2006, Australia; 2 School of Medicine, University of Notre Dame, Fremantle, WA 6959, Australia; lksurmon@outlook.com

**Keywords:** cryopreservation, spermatozoa, sperm cryopreservation, sperm DNA fragmentation, sperm plasmalemma, vitrification

## Abstract

Due to their negligible cytoplasm and composition of the sperm plasmalemma, spermatozoa are particularly vulnerable to lipid peroxidative damage induced by reactive oxygen species (ROS). Most ROS are referred to as free radicals because they have unpaired electrons, causing them to scavenge electrons from atoms within tissues, resulting in oxidative damage to cellular components including cell membranes, intracellular organelles, and DNA. The potential consequences of oxidative stress include impaired sperm function, DNA fragmentation, and apoptosis. Understanding the mechanisms that mediate sperm damage during cryopreservation is key to the development of improved sperm freezing media formulations and methodology to mitigate its occurrence. Historically, elucidation of those mechanisms has proven largely elusive and is complicated by the positive role that ROS also play as messengers in redox signaling and the different pathways that mediate sperm DNA damage and apoptosis. More recently, oxidative stress has been revealed as the most likely suspect in cryopreservation-induced sperm DNA damage. This narrative review was intended to provide an in-depth analysis of the mechanisms involved and offer insight into possible improvements in sperm cryopreservation.

## 1. Introduction

Assisted reproduction is a multidisciplinary branch of medicine requiring the coordinated efforts of specialists with different areas of expertise, including reproductive endocrinologists, anesthetists, gynecologists, urologists, andrologists, embryologists, clinical geneticists, and molecular cytogeneticists. However, precious few fertility centers benefit from the inclusion of cryobiologists among their staff, so expertise in the cryopreservation of reproductive tissues is relatively sparse. Nevertheless, cryopreservation is essential for the provision of a safe, efficient, and comprehensive clinical service for fertility patients. In this respect, it enables the donation and transport of reproductive tissues, including gametes, gonadal tissues, embryos, and stem cells. Also, it facilitates the incorporation of genetic testing of blastocysts and supports the practice of elective single embryo transfer to significantly reduce the risks of miscarriage and multiple pregnancy. Most importantly, perhaps, cryopreservation of reproductive tissues and gametes is the primary means of preserving fertility.

Human sperm cryopreservation has been an integral part of assisted reproduction since the advent of artificial insemination in the mid-20th century. Indeed, it is generally considered mandatory for semen to be quarantined in liquid nitrogen (LN_2_) to enable screening of prospective semen donors, ensuring they test negative for infectious diseases including Hepatitis B Virus (HBV), Hepatitis C Virus (HBC), and Human Immunodeficiency Virus (HIV) prior to use. The cryo-storage of human semen has encouraged the establishment of so-called ‘sperm banks’ around the world, which has increased access to fertility services for couples where the male is infertile and for women without a partner and those in same-sex partnerships. Precautionary semen cryopreservation may be advised in the event of diminishing sperm counts, prior to vasectomy, and where it may prove impossible to obtain a semen sample from a patient undergoing assisted reproduction with their partner, for example for individuals serving overseas in the armed forces. Furthermore, with the knowledge that sperm quality declines after the age of 35 and with the known link between paternal aging and the incidence of dominant genetic diseases in the offspring, there may be an imperative for younger men to cryopreserve their semen for future use. When faced with the necessity for gonadotoxic chemotherapy and/or radiotherapy treatment for various cancers, preservation of male fertility is entirely dependent upon prior cryopreservation of semen and testicular tissue. Similarly, potentially gonadotoxic treatment of some non-malignant diseases such as diabetes, glomerulonephritis, inflammatory bowel disease, and multiple sclerosis may also necessitate preservation of fertility [1,2,3,4]. Sperm cryopreservation is also a common ancillary to diagnostic surgical sperm retrieval (SSR) to avoid the risk of having to repeat a surgical procedure during a cycle of in vitro fertilization (IVF). Even in pre-pubertal boys, testicular tissue, testicular cell suspensions, and spermatogonial stem cells may be cryopreserved to preserve their fertility [5]. In extreme cases, testicular tissue may be cryopreserved post-mortem for potential conception by the surviving partner. Unfortunately, optimal sperm cryopreservation methodology remains elusive, and it is not uncommon for morphological, structural, and functional impairments of spermatozoa to occur due to cryopreservation [6]. Consequently, rates of cryo-survival may be low with, at best, only 50–60% of spermatozoa retaining their motility. Indeed, freezing and thawing of human semen may lead to increased generation of reactive oxygen species (ROS) and depletion of both intracellular and extracellular endogenous antioxidants, resulting in oxidative stress, increased lipid membrane peroxidation, and sperm DNA damage [7].

There has been much debate regarding the prognostic value and impact of sperm DNA quality upon laboratory and clinical outcomes, particularly following various means of assisted reproduction [8,9,10,11,12]. Of particular interest are the putative relationships between male and female age, sperm DNA damage and oocyte quality, and miscarriage [13,14]. Depending upon the quality of the oocyte’s DNA repair mechanism, the extent of single- and double-strand breaks in sperm DNA may have differential effects upon embryo viability, implantation, and ongoing pregnancy [15]. In this respect, single-stranded DNA breaks are related to oxidative stress and occur as multiple break points across the genome, leading to implantation failure. On the other hand, double-stranded breaks are more likely to be related to a lack of DNA repair during spermatogenesis and are more localized to the sperm nuclear matrix, resulting in poor embryo quality and miscarriage.

## 2. Reactive Oxygen Species and Oxidative Stress

One likely limitation to success in the in vitro alleviation of infertility is the oxidative stress that gametes and embryos are subjected to during various techniques performed outside of the body, including oocyte and sperm retrieval, sperm preparation, oocyte denudation, micromanipulation, embryo transfer, and cryopreservation. None of these procedures benefit from the controlled environment of an incubator where the oxygen tension is typically set much lower than that within the surrounding atmosphere. Indeed, it is a logical conclusion that increased exposure of gametes and embryos to the environment will result in cumulative oxidative stress, cryopreservation often representing the ultimate challenge to a cell following the sequential stresses of previous procedures. Under normal physiological conditions in vivo, homeostasis in the reduction–oxidation (redox) status of cells is maintained via a balance between endogenous antioxidants and oxidants. Indeed, a continuously shifting equilibrium in redox status is essential to intermediate signaling during normal cell function, but should this natural equilibrium be disrupted it can lead to cell pathology. Cells are endowed with internal defense mechanisms which can protect against oxidation including the antioxidants Vitamin C (ascorbic acid), Vitamin E (alpha-tocopherol), and Glutathione Peroxidase. Scavenging of oxygen radicals by enzymes, including Catalase, Peroxidase, and Superoxide Dismutase (SOD), is another means by which cells protect themselves from oxidative damage. Catalase and Peroxidase promote the decomposition of hydrogen peroxide (H_2_O_2_) to oxygen (O_2_) and water (H_2_O). Superoxide is converted by SOD into O_2_ or H_2_O_2_. Evidently, these defense mechanisms can act in concert to keep oxidative activity in check. However, if the antioxidant defense of cells becomes overwhelmed by supraphysiological levels of oxidation, then oxidative stress will ensue. Conversely, an over-response to oxidation or overuse of antioxidants can result in reductive stress, which can also render cells more vulnerable to infection [16,17]. The primary mediators of oxidative stress are a variety of molecules collectively referred to as ROS.

Molecules known as ROS are highly reactive chemicals derived from diatomic O_2,_ H_2_O and H_2_O_2_. Essentially, they are intrinsically unstable molecules containing oxygen and include products of aerobic metabolism such as H_2_O_2_, the hydroxyl radical (^.^OH), and the superoxide anion (O_2_^−^). Except for H_2_O_2_, most ROS have unpaired electrons and, therefore, are referred to as free radicals. Hence, by definition, free radicals tend to readily react with other molecules. Indeed, free radicals will readily scavenge electrons from other atoms present within cells, which can result in oxidative damage to various cellular components such as lipids, proteins, and DNA, as well as intracellular organelles including mitochondria. Intracellular, extracellular, and environmental triggers and sources of ROS may adversely impact cell function and viability. Endogenous sources of ROS include mitochondrial Oxidoreductase, membrane-bound enzyme complexes such as Nicotinamide Adenine Dinucleotide Phosphate Oxidase (NADPH oxidase), and enzyme activities such as Xanthine Oxidase (XO). Production of the O_2_^−^ free radical is catalyzed by NADPH oxidase via its transfer of an electron to oxygen. The activity of XO generates various ROS including H_2_O_2_ and superoxide ions. Exogenous triggers of ROS include environmental variables such as ambient oxygen tension, humidity, and light and the presence of metal ions including copper and iron. Also, increased levels of ROS have been associated with individual-specific factors including a sedentary lifestyle, diet and obesity, stress, consumption of alcohol, and smoking and recreational drug use [18]. Alarmingly, increased exposure to environmental pollution, including endocrine-disrupting chemicals, microplastics, and radiofrequency electromagnetic radiation, has also been identified as a potential trigger of ROS-induced oxidative stress [19]. Unsurprisingly perhaps, increased levels of ROS have been observed in several reproductive pathologies including endometriosis, intrauterine growth retardation, miscarriage, polycystic ovary syndrome, and preeclampsia [17].

A normal byproduct of cellular aerobic metabolism, the generation of ROS in spermatozoa is primarily mediated by electron leakage from the mitochondrial electron transport chain, cytosolic L-amino acid oxidases, or from the NADPH oxidase system, situated within the sperm plasmalemma [20]. However, it is the redox balance within spermatozoa which is relevant because at moderate levels ROS are necessary for the stability and protection of sperm DNA and normal sperm functions including capacitation, hyperactivation, the acrosome reaction, and fertilization of the oocyte [21]. Indeed, reductive stress and oxidative stress can both be detrimental to sperm function, so the correct balance must be maintained [22]. Under normal physiology, ROS are usually neutralized by the cell’s endogenous antioxidant system but can damage lipids, proteins, and nucleic acids if allowed to reach critical levels. Indeed, a vicious cycle of oxidative DNA damage can arise, in which ROS-induced lipid peroxidation releases lipid aldehydes which can bind to proteins in the mitochondrial electron transport chain, leading to further production of ROS [23,24]. With respect to sperm preparation, ambient temperature, incubation at 37 °C, and excessive centrifugation are additional potential triggers of ROS generation [25].

## 3. The Impact of Reactive Oxygen Species upon Spermatozoa

Due to their limited cytosol, high levels of polyunsaturated fatty acids in their cell membranes; lack of endogenous, cytoplasmic, antioxidant enzymes; and DNA repair mechanisms, spermatozoa are particularly vulnerable to peroxidative damage by ROS. Therefore, sperm structural and functional integrity may be impaired by oxidative stress, especially during cryopreservation. Toxic byproducts of lipid peroxidation, 4-Hydroxynonenal (4-HNE), Malondialdehyde (MDA), and Acrolein, disrupt the sperm plasmalemma and mitochondrial proteins of the electron transport chain [26]. Lipid peroxidation caused by oxidative stress results in reduced sperm plasmalemma fluidity and integrity, which can impair sperm motility, capacitation, the acrosome reaction, and fertilization [20,27]. Metabolic disruption of the mitochondrial electron transport chain can adversely impact sperm quality via increased production of mitochondrial ROS [28]. Mitochondrial membrane damage can also adversely impact sperm motility [29]. Furthermore, ROS can damage sperm chromatin and DNA and, thereby, negatively impact male fertility [30]. The oxidative stress of ROS upon sperm DNA integrity is characterized by the generation of the DNA base adduct 8-Hydroxy-2′-Deoxyguanosine (8OHDG), the first enzyme in the base excision repair (BER) pathway, 8-Oxoguanine DNA Glycosylase 1 (OGG1) being the sperm’s only DNA repair mechanism [24]. Cryoinjury to sperm may be mediated by various mechanisms, including accidental cell death, due to extracellular and/or intracellular ice formation, intrinsic or extrinsic programmed cell death or apoptosis, and non-apoptotic regulated cell death including ferroptosis and necroptosis, as well as oxidative stress. These pathways of cryo-induced sperm DNA fragmentation overlap and interact to some extent, oxidative stress being able to trigger one or more forms of regulated cell death, and vice versa [16]. Accidental cell death during cryopreservation is discussed further in the Section 4 below.

**Figure 1:**
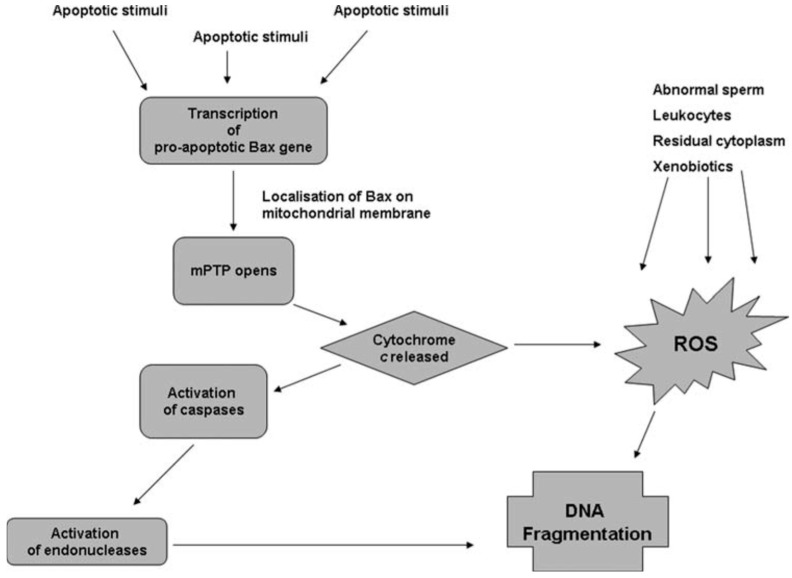
Pathways proposed to lead to sperm DNA fragmentation [41].

Apoptosis is regulated by products of lipid peroxidation which can activate the Nuclear Factor Kappa B, Mitogen-Activated Protein Kinase, and Protein Kinase C signaling pathways. Sperm cryodamage may be characterized by various markers of apoptosis including activation of caspases, externalization of phosphatidylserine, and a reduction in mitochondrial membrane potential [31]. Interestingly, though apoptosis is a normal feature of spermatogenesis, incomplete or abortive apoptosis occurs in response to the induction of sperm DNA fragmentation by endonucleases and may be responsible for the appearance of DNA-damaged spermatozoa within the ejaculate [32]. Caspases, which are specific aspartic acid-directed cysteine proteases, are known to play a role in the stress-induced apoptotic cascade and eventual cell death [33]. Indeed, correlations have been drawn between caspase activity, apoptotic markers, and sperm parameters within ejaculated human semen [34,35]. Activation of caspases has been observed in human spermatozoa following cryopreservation, and the presence of glycerol in the sperm freezing medium may exacerbate this phenomenon [36,37]. The differential relationships between oxidative stress and apoptosis with sperm DNA damage can be illustrated by the concurrent pathways believed to be involved in sperm DNA fragmentation (Figure 1). In the apoptosis pathway, apoptotic stimuli trigger the transcription of pro-apoptotic genes, including *Bax*, which encodes the mitochondrial membrane’s pro-apoptotic Bax protein [38]. Bax can open the mitochondrial permeability transition pore, resulting in mitochondrial swelling and release of Cytochrome C [39]. Once Cytochrome C enters the cytoplasm, it activates caspases which activate specific endonucleases, causing sperm DNA fragmentation [38]. As shown in Figure 1, Cytochrome C can also cause DNA fragmentation via increased production of ROS and oxidative DNA damage [40]. Hence, in the ejaculate at least, ROS generated by abnormal spermatozoa and non-germ cells may compound any DNA fragmentation caused by the apoptotic cascade. On the other hand, sperm DNA fragmentation appears to occur independently of caspase activation in cryopreserved semen, presumably within non-apoptotic spermatozoa [41].

It is well understood that ROS may be a cause of oxidative stress to spermatozoa, which provides an alternative mechanism for possible induction of sperm DNA damage by cryopreservation [25,42]. Indeed, the superoxide anion, which is capable of spontaneously or enzymatically undergoing a redox reaction to yield H_2_O_2_, is well-known to be generated by human spermatozoa [43,44]. In this respect, H_2_O_2_ is considered especially cytotoxic as it can cross the cell membrane and has high activity as an oxidant. Furthermore, the extremely noxious hydroxyl radical, which can attack almost every cellular component, is a product of superoxide and H_2_O_2_ [45]. Under normal circumstances, spermatozoa are protected from oxidative stress by the presence of enzymatic and non-enzymatic antioxidants within the seminal plasma [21]. These include oxygen radical scavengers such as Catalase and Peroxidase, which promote the breakdown of H_2_O_2_ into O_2_ and H_2_O—and SOD. However, particularly following removal of seminal plasma during sperm preparation, generation of ROS during sperm cryopreservation may overwhelm endogenous antioxidants, resulting in oxidative stress and DNA damage [46]. Indeed, the generation of ROS during sperm cryopreservation has been observed in several species, suggesting a relationship between oxidative stress and cryoinjury to sperm DNA [47,48,49,50]. The oxidative stress experienced by spermatozoa is compounded by cryopreservation since absorption of their cytoplasm reduces their reserves of enzymatic and ROS-scavenging antioxidants, and metabolic recovery during freezing and thawing causes a surge in their endogenous levels of ROS. Interestingly, semen cryopreservation may be especially detrimental to the spermatozoa of male factor infertility patients [51,52]. Furthermore, any spermatozoa with already compromised chromatin structure and DNA integrity may prove particularly susceptible to the oxidative stress of cryopreservation [24,53]. Lipid peroxidation of the sperm plasmalemma by ROS and the resulting oxidative stress is also intricately linked to other pathways of regulated cell death. One of these pathways, termed ferroptosis, is characterized by a lethal level of widespread, iron-dependent, lipid peroxidation [54]. Cell protection against ferroptosis is achieved by scavenging and reduction in lipid peroxides, particularly by Coenzyme Q10 (CoQ_10_), which is abundant throughout the cell membrane [55]. Another pathway like apoptosis is necroptosis, which is characterized by morphological features typical of cell necrosis and has been associated with increased exposure of the cell to H_2_O_2_ and inactivation of Caspase 8 [56]. Interestingly, Necrostatin-1 which inhibits activation of Receptor Interacting Protein 1 (RIP1), a key mediator of necroptosis, can significantly improve the cryopreservation of spermatogonial stem cells [57].

## 4. Sperm Cryopreservation and Sperm Cryoinjury

Basic research in cryobiology has led to the development of various methods for the cryopreservation of semen, spermatozoa, and testicular tissue, including dry ice pelleting, LN_2_ vapor freezing, rate-controlled slow freezing, and ultra-rapid cooling or vitrification. These techniques mostly rely upon the incorporation of various permeating and non-permeating cryoprotective agents (CPAs) to avoid or minimize sperm cryoinjury during both cooling/freezing and warming/thawing procedures. In this respect, the primary objective is to preserve sperm viability, membrane function, and motility. The main approaches to sperm cryopreservation are slow freezing, rapid freezing, and vitrification. Slow or controlled-rate freezing employs either passive manual exposure to LN_2_ vapor or a computer-controlled device to control a pre-programmed decrease in temperature over an extended period. Rapid freezing is typically achieved via direct contact between sperm straws and LN_2_ vapor for 8–10 min, followed by direct immersion in LN_2_ to rapidly reduce the temperature to −196 °C. Like rapid freezing, vitrification also relies upon direct immersion into LN_2_. Unlike slow freezing and rapid freezing, vitrification is better suited to cryopreservation of small numbers of spermatozoa, such as those retrieved from the epididymis and testis via SSR.

With slow freezing, spermatozoa may suffer extensive chemical and physical damage, most likely due to intracellular ice crystallization. Primarily, this is because of the water to ice transition phase that occurs between −15 °C and −60 °C, which spermatozoa must survive during both freezing and thawing [58]. Prior to that, during the cooling phase, cells and tissues lose their osmotic equilibrium with the freezing medium which freezes at a higher temperature (−5 °C) than the cells, which have a lower freezing point due to the salts within their cytoplasm. Cells become supercooled between −5 °C and −10 °C, and extracellular ice formation increases, resulting in an increased extracellular solute concentration. This forces cells to osmotically equilibrate with the extracellular environment by rapidly losing water, leading to severe dehydration and shrinkage, which increases the risk of osmotic shock. Cells may then be subjected to additional physical damage due to the ‘packing effect’ of increasing cell–ice and cell–cell contacts that occur between −10 °C and −15 °C, following the expansion of the extracellular ice. By this stage, further osmotic equilibrium is achieved via the assembly of trans-membrane ice crystal–hydrogen bonds, resulting in intracellular freezing. Furthermore, during thawing spermatozoa may again be subjected to osmotic shock due to excessive cellular swelling [59]. Indeed, it has been suggested that sperm cryoinjury occurs predominantly during thawing due to loss of antioxidant defense activity and structural damage to the cytoskeleton [60,61]. Hence, the major threats to sperm cryo-survival are osmotic shock and intracellular ice formation.

The damage that cells and tissues might otherwise suffer during cryopreservation may be modulated to some extent by CPAs, which are organic solutes that modulate the phase transition between water and ice [62]. Primarily, CPAs prevent excessive cell shrinkage and inhibit intracellular and extracellular ice formation during freezing but also help preserve cell membranes and promote vitrification. By virtue of their molecular size and ability to diffuse through cell membranes, CPAs occupy either the intracellular or extracellular space, cell membranes generally only being permeable to smaller molecules such as dimethyl sulfoxide (DMSO), glycerol, and 1,2-propanediol, also known as propylene glycol. Serendipitously in 1949, glycerol, ethylene glycol, and propylene glycol were the first CPAs discovered to improve human sperm cryo-survival following vitrification, and they remain in common usage [63]. Ethylene glycol, glycerol, and 1,2-propanediol are kosmotropic, meaning that they possess anti-freeze properties, forming strong hydrogen bonds with water, thereby competing with the hydrogen bonds connecting water molecules. Hence, they effectively disrupt the potential for intracellular ice formation, specific alignment of water molecules being necessary for crystalline ice formation. Unfortunately, the ability of cell-permeable CPAs to prevent intracellular ice formation depends upon a slow cooling rate which can be detrimental to sperm viability per se. Used to reduce ice formation in diverse applications, DMSO is an aqueous, hydrogen bonding, polar organosulfur compound, with very high permeability through the cell membrane. Cell-permeable CPAs have various mechanisms of action; they reduce the temperature at which cells freeze and raise the temperature at which they vitrify, and some increase cell membrane permeability, which facilitates cell dehydration. Some cell-permeable CPAs prevent membrane damage by inhibition of adjacent cell membrane fusion via interaction with phospholipids within the lipid bilayer. Cell-permeable CPAs also protect intracellular organelles. These cell-permeable cryoprotectants all have a molecular mass below 100 g.mol^−1^. In contrast the disaccharides, sucrose and trehalose, have a molecular mass of 342.3 g.mol^−1^ and 342.296 g.mol^−1^, respectively and, therefore, are unable to diffuse across the cell membrane.

Non-permeating CPAs influence the osmolality of the extracellular environment, thereby modulating osmosis during cellular dehydration and rehydration. Generally, at the same molar concentrations, non-permeating CPAs are less toxic to cells than permeating CPAs and reduce the concentration of permeating CPAs required for vitrification. During cooling, non-permeating CPAs increase cellular solute concentration via dehydration and increase carrier solution viscosity, thereby promoting colligative freezing point depression and vitrification, respectively. Non-permeating CPAs are also believed to adsorb to the outer cell membrane, thereby protecting the cell from extracellular crystal lattice ice formation. Furthermore, non-permeating disaccharides lower the lipid membrane’s phase transition temperatures via non-specific osmotic and volumetric effects, thereby helping maintain cell membrane integrity.

The intrinsic response of spermatozoa to cryopreservation is likely to vary depending upon whether they are within seminal plasma, in cell suspension, or embedded within other cell types present within the testis. Uniformly rapid cooling and warming rates are evidently more difficult to achieve within complex tissues. Intuitively, it may seem preferable to cryopreserve the entire ejaculate since the viability and cryo-stability of mitochondrial and sperm cell membranes is supported by lipoproteins within the seminal plasma [64]. Furthermore, it has been suggested that removal of seminal plasma brings about premature capacitation, sperm membrane instability, and a reduction in the viable lifespan of spermatozoa [65]. Seminal plasma proteins also possess antioxidant properties [66,67]. This concept has been supported by several studies reporting that freezing prior to preparation of semen results in significantly increased viable, progressively motile sperm counts [67,68]. However, this practice has been questioned recently, with density gradient centrifugation (DGC) preparation of semen prior to rate-controlled slow freezing having been shown to yield a highly significant increase in the total number of viable, progressively motile spermatozoa following thawing [69]. Indeed, this is a debatable issue, with other studies also supporting the pre-preparation approach to sperm freezing [70,71,72,73,74]. A possible explanation for these observations is that removal of seminal plasma increases sperm membrane fluidity, thereby increasing permeability to CPAs to maximize cryoprotection [75]. Hence, the relationship between semen preparation and sperm cryopreservation appears to be a complex one, with optimization of approach being complicated by the need to balance access of CPAs with maintenance of the sperm membrane’s architectural integrity.

Ultra-rapid liquid solidification or vitrification of spermatozoa may mitigate the risk of intracellular ice formation to some extent, plunging into the LN_2_ avoiding phase transition damage [76]. Vitrification may also be achieved in the absence of potentially toxic cell-permeable CPAs [77]. Indeed, successful vitrification may be achieved using only non-permeating compounds such as human serum albumin and sucrose by virtue of their antioxidant and osmotic qualities, respectively. This method has been used successfully for even large volumes of semen in the absence of any CPAs [78]. With respect to sperm DNA integrity, vitrification in the absence of CPAs compares favorably against cryopreservation using a wide range of commercially available sperm freezing media [53]. Indeed, there appears to be no detrimental impact of cryoprotectant-free vitrification upon sperm DNA fragmentation and hyaluronan binding when compared with rapid freezing and freezing using cell-permeable CPAs [79]. Interestingly, even vitrification in the absence of both sucrose and cell-permeable CPAs has been reported to yield higher sperm viability and lower cryodamage to sperm chromatin and sperm DNA [80,81]. However, the benefit of cryoprotectant-free vitrification of human semen is not a universal finding, with some authors recommending vitrification of spermatozoa following separation from seminal plasma [82].

During cryopreservation, spermatozoa are subjected to oxidative stress via consumption of antioxidants due to sperm cytosol absorption and disruption of the extracellular environment, which destabilizes sperm metabolism and increases the levels of ROS. Unfortunately, there has been little evolution in the composition of human sperm freezing media for decades, and there is clearly a need for greater understanding of the mechanisms involved in sperm DNA damage during cryopreservation. Further research in this area will, hopefully, lead to improvements in cryomedia and cryopreservation techniques.

## 5. Antioxidants and Their Mitigation of Sperm Cryodamage

Oral antioxidant therapy for men suspected of being exposed to oxidative stress has proven to be of limited value to their semen quality and fertility [83]. Unfortunately, this area of research has been largely undermined by a general failure to effectively diagnose those suffering from oxidative stress and, therefore, identify patient groups most likely to benefit from administration of oral antioxidants [84]. Furthermore, it has been pointed out that little value in the administration of antioxidants can be expected in the absence of a deficit in endogenous antioxidant activity [84]. Conversely, there can be little doubt that spermatozoa will be subjected to oxidative stress during various procedures conducted in vitro. Indeed, a range of antioxidants such as Catalase, CoQ10, Ethylenediaminetetraacetic Acid (EDTA), Melatonin, and vitamins have been shown to improve sperm parameters during various in vitro processes [85,86]. Interestingly, a multi-antioxidant approach may better address the diversity of the redox systems known to operate within spermatozoa, a synergistic effect of various antioxidants during cryopreservation having been demonstrated in the animal model [87,88,89,90]. Therefore, it is likely that a similar approach might be beneficial to the cryopreservation of human semen and spermatozoa.

**Figure 2:**
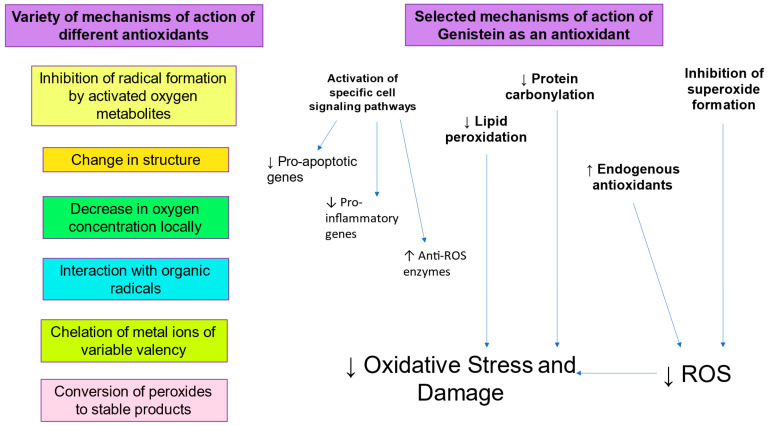
Role of antioxidants in mitigating oxidative stress.

There is clearly an imperative for the development and clinical application of more effective cryomedia and cryopreservation protocols [91]. In this respect, the inclusion of antioxidants such as Genistein to prevent sperm DNA damage due to oxidative stress during cryopreservation would be a logical strategy (Figure 2). Genistein, a phyto-estrogen derived from soyabeans, has been found to protect against oxidative stress, lipid peroxidation, and resulting sperm DNA fragmentation [41]. Genistein appears to have antioxidant activity since it can protect cells from oxidative stress, lipid peroxidation, and DNA fragmentation [92,93]. Its mechanism of action includes inhibition of enzymes capable of generating ROS, such as XO, inhibition of lipid peroxidation by scavenging of peroxyl radicals, and enhancement of the activity of antioxidants endogenous to spermatozoa, such as Glutathione Peroxidase, Glutathione Reductase, and SOD [94,95]. Interestingly, Genistein also displays anti-mutagenic activity and can inhibit ROS-induced DNA fragmentation [96,97]. Indeed, Genistein has a significant protective effect against oxidative DNA damage and sperm DNA fragmentation, suggesting that increased oxidative stress generates sperm DNA damage during cryopreservation [41]. Sperm vitality and post-thaw motility is also improved by the presence of Genistein, suggesting a link between oxidative stress and sperm function.

Various other enzymatic and non-enzymatic antioxidants have been shown to be beneficial to sperm function and DNA integrity [98]. Hypotaurine and the powerful antioxidant, Glutathione, have been shown to protect against H_2_O_2_-induced sperm DNA damage [99]. Interestingly, Hypotaurine also has a protective effect against apoptosis, but only when sperm are separated by DGC prior to freezing [100]. The antioxidants, Vitamin C and Vitamin E, also protect against sperm DNA damage during sperm preparation [101,102]. Other studies of Vitamin C and Vitamin E have reported a reduction in cryopreservation-induced ROS production and increase in post-thaw motility, respectively [103]. Edaravone is a low-molecular-weight antioxidant which scavenges both lipid- and water-soluble peroxyl radicals by donating an electron to them [104]. Other novel antioxidants merit further research. For example, the nucleophilic thiol, Penicillamine, has been shown to protect against oxidative stress in several studies involving human spermatozoa. In one of these studies, Penicillamine was able to protect against oxidative stress induced by Thiostrepton-mediated inhibition of the antioxidant enzymes 2-Cys Peroxiredoxins [105]. A mechanistic study of Penicillamine demonstrated that its protective effects against oxidative stress were mediated by a stabilization of 4-HNE generation and a decline in 8OHDG formation, affording protection of thiol groups within spermatozoa [106]. In another study, Penicillamine was shown to protect against Ionomycin and H_2_O_2_-induced oxidative stress in normozoospermic donor semen samples [107]. When added to the sperm incubation medium containing Ionomycin or H_2_O_2_, Penicillamine was able to reduce levels of ROS, resulting in improved preservation of mitochondrial membrane potential, ATP levels, and sperm motility. The amino acids, Cysteine and Glutamine, can also serve as effective antioxidants, decreasing intracellular ROS and sperm DNA damage, resulting in a significant improvement in sperm plasmalemma integrity, mitochondrial membrane potential, and progressive sperm motility [108]. Another naturally occurring antioxidant, Astaxanthin, has been found to decrease the levels of ROS and increase human sperm motility and DNA integrity following both slow freezing and vitrification [109,110,111].

## 6. Discussion

The cryopreservation of semen, spermatozoa, and testicular tissue is clearly not a stress-free process, which is hardly surprising given that the various procedures are performed under atmospheric conditions, with oxygen tensions of 20–21%. So, by definition, it is likely that spermatozoa will be subjected to oxidative stress. Though correlations between sperm DNA fragmentation and apoptosis have been reported previously, the evidence is not that strong and, besides, there is a paucity of evidence for an increase in sperm DNA fragmentation mediated by a caspase cascade during cryopreservation [112,113]. Though increased sperm DNA damage following cryopreservation has been extensively reported previously [7,20,23,24,114], there are relatively few studies demonstrating an association between oxidative base damage and sperm DNA fragmentation [41,112]. Supporting a link between cryo-induced oxidative DNA damage and sperm DNA fragmentation, there have also been reports of increased ROS production during sperm cryopreservation [47,48]. Since oxidative stress, sperm DNA fragmentation, and apoptosis occur within spermatozoa regardless of whether semen samples have been separated by DGC before or after cryopreservation, it appears that cryoinjury is suffered by both good- and poor-quality spermatozoa [113]. Notably, there appears to be a commonality between the mechanisms mediating the generation and exacerbation of oxidative DNA damage and sperm DNA fragmentation during cryopreservation [114].

Given that there are at least two distinct putative mechanisms of cryoinjury to sperm DNA, we designed a study to possibly differentiate between them using semen samples from 60 patients tested for sperm DNA fragmentation, oxidative DNA damage, and apoptosis, before and after cryopreservation [41]. Aliquots of each liquefied semen sample, with or without prior DGC preparation, were frozen by rate-controlled, slow freezing in either the absence (*n* = 20 controls) or presence of the soyabean-derived, estrogenic antioxidant Genistein (*n* = 20) or the cell-permeable, pan-caspase irreversible inhibitor, Z-VAD(OMe)-FMK (*n* = 20). Prior to freezing, a highly significant positive correlation was found between oxidative DNA damage and sperm DNA fragmentation in semen samples before (*p* < 0.001) and after (*p* < 0.01) DGC, a similar positive correlation (*p* < 0.001) between sperm DNA fragmentation and the percentage of caspase positive cells only being observed in the DGC separated fraction. Cryopreservation in the absence of Genistein and Z-VAD(OMe)-FMK resulted in a significant increase in oxidative DNA damage, sperm DNA fragmentation, and apoptosis, regardless of whether the semen samples were separated by DGC. Genistein addition prior to freezing was found to have a significant protective effect following thawing against oxidative DNA damage and sperm DNA fragmentation, resulting in a significant increase in sperm motility and vitality. In contrast, Z-VAD(OMe)-FMK addition prior to freezing was unable to provide any protective effect against oxidative DNA damage and sperm DNA fragmentation following thawing. These results suggest that the damage to sperm DNA induced during cryopreservation is predominantly mediated by oxidative stress rather than apoptosis, offering some potential opportunity to improve sperm cryopreservation media.

Since our study, there have been reports from other groups supporting our findings. To differentiate between the impact of sperm cryopreservation on single- and double-stranded DNA fragmentation, an analysis of the fresh and frozen semen samples from 30 males was conducted using alkaline and neutral Comet assays, respectively [115]. A 10% increase in single-stranded DNA fragmentation was observed following cryopreservation, regardless of male fertility status, whereas there was no significant increase in double-stranded DNA fragmentation. These results led the authors to conclude that oxidative stress is the major effector of sperm DNA damage since single-stranded sperm DNA fragmentation has been previously correlated with oxidative damage [116,117]. Another study compared the levels of apoptosis, oxidative stress, and sperm DNA damage in 60 infertility patients and 20 normozoospermic sperm donors prior to and following slow freezing of their semen samples, using an Annexin V-FITC apoptosis kit, BIODIPY/8OH-DG, and TUNEL staining, respectively [118]. The sperm donors were found to have significantly lower levels of apoptosis, oxidative stress, and DNA damage than those undergoing fertility treatment, and BIODIPY/8OH-DG levels were significantly higher than Annexin V levels post-thawing, suggesting the greater relevance of oxidative stress during cryopreservation.

## 7. Future Directions

The relative benefit of antioxidants during cryopreservation may be species-specific, tissue specific, and/or depend to some extent upon the freezing method employed. For example, the universal antioxidant, α-Lipoic Acid, which acts as a cofactor for mitochondrial enzyme activity and is capable of regenerating other antioxidants such as Glutathione, has been shown to protect against cryo-induced sperm DNA damage and improve sperm motility and acrosome integrity in boar spermatozoa [119]. On the other hand, with simple sucrose vitrification of semen samples from normozoospermic sperm donors, triple antioxidant (Acetyl-L-Carnitine, N-Acetyl-L-Cysteine, and α-Lipoic Acid) supplementation of the vitrification medium did not decrease extracellular levels of ROS and was found to have only a moderate, non-significant, protective effect against sperm DNA fragmentation [120]. This conundrum serves to illustrate the challenge in developing improved methods for sperm cryopreservation in the human and other mammalian species, given the confounding variables of sperm source, sperm preparation, sperm freezing medium, and cryopreservation technique. Furthermore, it may be unrealistic to expect antioxidants that act by increasing the activity of endogenous, enzymatic, antioxidant pathways within the limited sperm cytosol to be as effective as those that scavenge ROS. To complicate matters further, there are other groups of molecules related to ROS that may also be involved in cryoinjury to spermatozoa including reactive halogen (bromine and chlorine) species, reactive nitrogen species, and reactive sulfur species. Also, there are additional, oxidative stress-derived, regulated, cell death pathways which merit further research such as cuproptosis, where excessive accumulation of copper within the cell can cause metabolic disturbances. There exists precious little published data exploring these alternative pathways during sperm cryopreservation.

## 8. Conclusions

Spermatozoa are particularly vulnerable to oxidative stress by virtue of the high cellular content of polyunsaturated fatty acids within the sperm plasmalemma and limited reserves of antioxidants within their residual cytoplasm following spermiogenesis. Furthermore, timely genomic responses to environmental stress, such as the maintenance of ATP synthesis and redox homeostasis, are rendered impossible by the highly condensed chromatin within the sperm nucleus. Semen, spermatozoa, and testicular tissue are exposed to oxidative stress during cryopreservation, cryoinjury of sperm DNA predominantly occurring via the ROS pathway rather than via the apoptotic cascade. Therefore, vitrification and the addition of antioxidants to sperm cryomedia may mitigate the increased sperm DNA fragmentation observed following freezing and thawing.
